# A readily available alternative to Chinese finger traps for fracture reduction

**DOI:** 10.1308/003588413X13511609958055h

**Published:** 2013-03

**Authors:** KSN Akhtar, D Akhtar, J Simmons

**Affiliations:** Imperial College Healthcare NHS Trust, UK

## Background

Closed reduction of forearm and hand fractures can be performed with Chinese finger traps.^1^ These are applied individually to the fingers and the limb is suspended, with gravity providing countertraction to disimpact the fracture by ligamentotaxis. The fracture fragments are manipulated once length is restored. Chinese finger traps are a valuable tool that can maintain traction while a cast is applied and can also be useful during fracture fixation.^2^ They are not readily available in many hospitals, however, particularly out of hours.

## Technique

**Figure 1:**
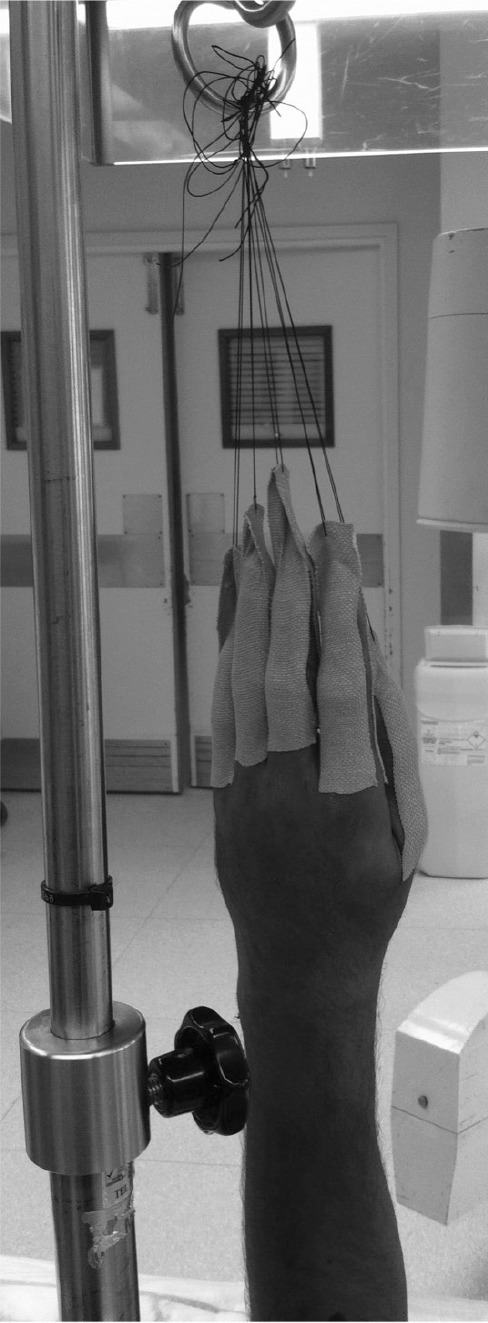
Modified finger traps

Strips of Tensoplast^®^ tape (2.5cm × 4.5m; BSN medical, Hull, UK) are placed over the thumb and each finger in turn. A strip is placed over the dorsal and palmar aspects of each digit, ensuring that the metacarpophalangeal joints are covered to prevent shearing and blister formation. Approximately 1 inch of Tensoplast^®^ is left loose distal to the fingertip and pinched together (Fig 1). A silk suture is passed through each of these loose ends, the needle is removed and the resulting silk loops are tied to a drip stand.

## Discussion

This technique ensures that each digit is suspended individually in a similar manner to Chinese finger traps. Gravity can be used to facilitate ligamentotaxis and fracture reduction. It is our practice to place a padded arm board below the arm as a ‘safety net’ but we have not experienced a hand falling out of this arrangement. This technique is particularly useful if there is no assistant available.
